# Addiction Recovery Among Opioid-Dependent Patients Treated With Injectable Subcutaneous Depot Buprenorphine: Study Protocol of a Non-randomized Prospective Observational Study (ARIDE)

**DOI:** 10.3389/fpsyt.2020.580863

**Published:** 2020-12-08

**Authors:** Bernd Schulte, Kirsten Lehmann, Christiane Sybille Schmidt, Elke Rühling, Bernd Weber, Ingo Schäfer, Jens Reimer, Uwe Verthein

**Affiliations:** ^1^Department of Psychiatry, Centre for Interdisciplinary Addiction Research of Hamburg University (ZIS), University Medical Center Hamburg-Eppendorf, Hamburg, Germany; ^2^Praxiszentrum Friedrichsplatz, Competence Center for Addiction Medicine, Kassel, Germany; ^3^Gesundheit Nord, Bremen, Germany

**Keywords:** addiction recovery, opioid substitution treatment (OST), injectable depot buprenorphine, quality of life, social participation

## Abstract

**Introduction:** Once-weekly or once-monthly injectable depot buprenorphine is a new opioid substitution treatment (OST) medication that provides clinically relevant plasma concentrations without daily peaks. Together with a high tolerability and acceptance reported by patients, the prolonged release of injectable depot buprenorphine might have beneficial implications on the patients' quality of life and social participation. The primary objective of this prospective non-interventional observational study is to evaluate the effects of subcutaneous injectable depot buprenorphine on the quality of life of patients in routine OST care in Germany. Secondary outcomes like illicit substance use, psychological distress, social participation and activity are assessed to provide an overall evaluation toward addiction recovery.

**Methods and Analysis:** The present study is a non-randomized prospective observational study with a control group (treatment-as-usual). To ensure comparability between both patient groups, suitable control patients (*n* = 213) from the same OST unit will be matched pairwise to each patient treated with injectable depot buprenorphine (*n* = 213). Matching variables are gender, duration of OST, take-home prescription and psychosocial functioning (according to the Global Assessment of Functioning scale). Primary study endpoint is the difference of change in quality of life, assessed with the recently developed Opioid Substitution Treatment Quality of Life scale (OSTQOL), within the depot buprenorphine group between baseline and month 12. The primary analysis will be carried out according to the intention-to-treat principle (ITT) by comparing OSTQOL mean scores using dependent *t*-tests. For secondary analyses, group comparisons will be done by mixed model approaches with baseline OSTQOL score and the (pairwise) cluster term as covariates.

**Discussion:** The study combines clinical, routine OST care data with relevant patient reported outcome data. The pairwise matching allows conclusions on effects of different OST medications. The study findings will provide new insights in the addiction recovery processes of OST patients treated with depot buprenorphine.

**Ethics and Dissemination:** The study protocol has been approved by the Ethics Committee of the Hamburg Chamber of Physicians (Ärztekammer Hamburg) (reference number: PV7078). The study results will be disseminated through peer-reviewed publications and presentations on scientific conferences.

**Clinical Trial Registration:** German Clinical Trials Register DRKS-ID: DRKS00020797

## Introduction

Opioid dependence is a chronic relapsing disease that is associated with a high health burden and enormous social consequences for the patient (1). In terms of accessibility, retention and effectiveness, opioid substitution treatment (OST) has become the primary option for the treatment of opioid dependence in Germany. As of July 1st, 2019, 79,700 opioid-dependent persons in Germany were registered in OST (2). While OST decreases drug-related deaths and reduces illicit opioid use, the effectiveness of OST remains limited with regard to the other objectives of the respective German Medical Association guidelines (3), e.g., abstinence from opioids, the stabilization of health, and an improvement in social participation (1, 4). The rate of OST patients in Germany who regularly terminate OST with opioid abstinence is low (1, 4–6) and the health and social burden remains high among many patients even after years in OST (1, 4, 7). Given the wide-ranging health and social impact of opioid dependence and the limitation of traditional health parameters to reflect the complexity and severity of the condition, patient-related outcome measures (PROMs) such as quality of life are needed to measure the holistic and integrative approach of OST (8–10) and to inform about the course of treatment and addiction recovery processes within OST (11).

Clinical trials have shown that the sustained release of the newly approved once-weekly or once-monthly injectable subcutaneous depot buprenorphine formulations produce an immediate and sustained opioid blockade and efficiently suppress opioid withdrawal after the first injection (12, 13). After the subcutaneous injection the depot buprenorphine transforms into a liquid-crystalline gel matrix that encapsulates buprenorphine and releases it in controlled and steady rates. The gel-like depot is biologically degraded over time, which minimizes the initial burst-like release of oral buprenorphine (14–16). Depot buprenorphine with different doses and dosing frequencies enables more flexible and individualized therapies of opioid use disorders across all treatment stages, from initiation and stabilization to long-term maintenance and preparation for regular OST termination.

A pharmacokinetic evaluation shows clinically relevant plasma concentrations of buprenorphine without daily peaks, which suggests potential benefits of injectable depot buprenorphine in the avoidance of withdrawal symptoms (17). Accordingly, patients reported higher acceptability of injectable depot buprenorphine compared with the sublingual formulation (17). The long-acting formulation together with pharmacokinetic profiles and the high tolerability and acceptance reported by patients indicate that injectable depot buprenorphine can have beneficial implications on the patients' adherence as well as quality of life (17).

Previous literature compared changes in quality of life between depot buprenorphine and placebo (18, 19). Compared with placebo, patients receiving extended-release injectable buprenorphine showed significantly improved patient-reported outcomes with regard to health and medication satisfaction after 6 months (18). Moreover, their employment rate after 6 months increased, whereas the number of hospital days per person-year decreased (18). The 12-month follow-up confirmed that OST with subcutaneous injected buprenorphine can result in high retention and abstinence rates as well as in improved social aspects like employment (19).

In contrast to previous literature, this study will take a more naturalistic approach, by comparing not only against placebo, but against treatment as usual (TAU), which includes all registered OST medications, such as methadone or levomethadone, sublingual buprenorphine or slow-release oral morphine. By comparing against other forms of OST, including sublingual buprenorphine, we aim to evaluate the potential advantages that are specifically related to the depot formulation, e.g., the decrease of doctor-patient contacts, which might support patients' independence and social participation. In addition to generic quality of life and mental health instruments, we will use the new Opioid Substitution Treatment Quality of Life scale (OSTQOL) (20) as our main outcome measure, to account for the whole range of aspects of recovery that are relevant for opioid-dependent patients (20).

## Methods and Analysis

### Objectives

The primary objective of this study is to evaluate the quality of life among patients substituted with subcutaneous injectable depot buprenorphine. Secondary objectives include treatment satisfaction, acceptance of injectable depot buprenorphine and its application form, illicit substance use, retention in treatment, craving, psychological distress, social participation and activity, and cost-effectiveness. The primary and secondary objectives are selected to provide an overall assessment toward addiction recovery.

### Hypothesis

OST with once-weekly or once-monthly injectable depot buprenorphine will result in increased quality of life, as measured by the Opioid Substitution Treatment Quality of Life scale (OSTQOL) (20) between baseline (t_0_) and month 12 (t_4_).

### Study Design

**Figure 1:**
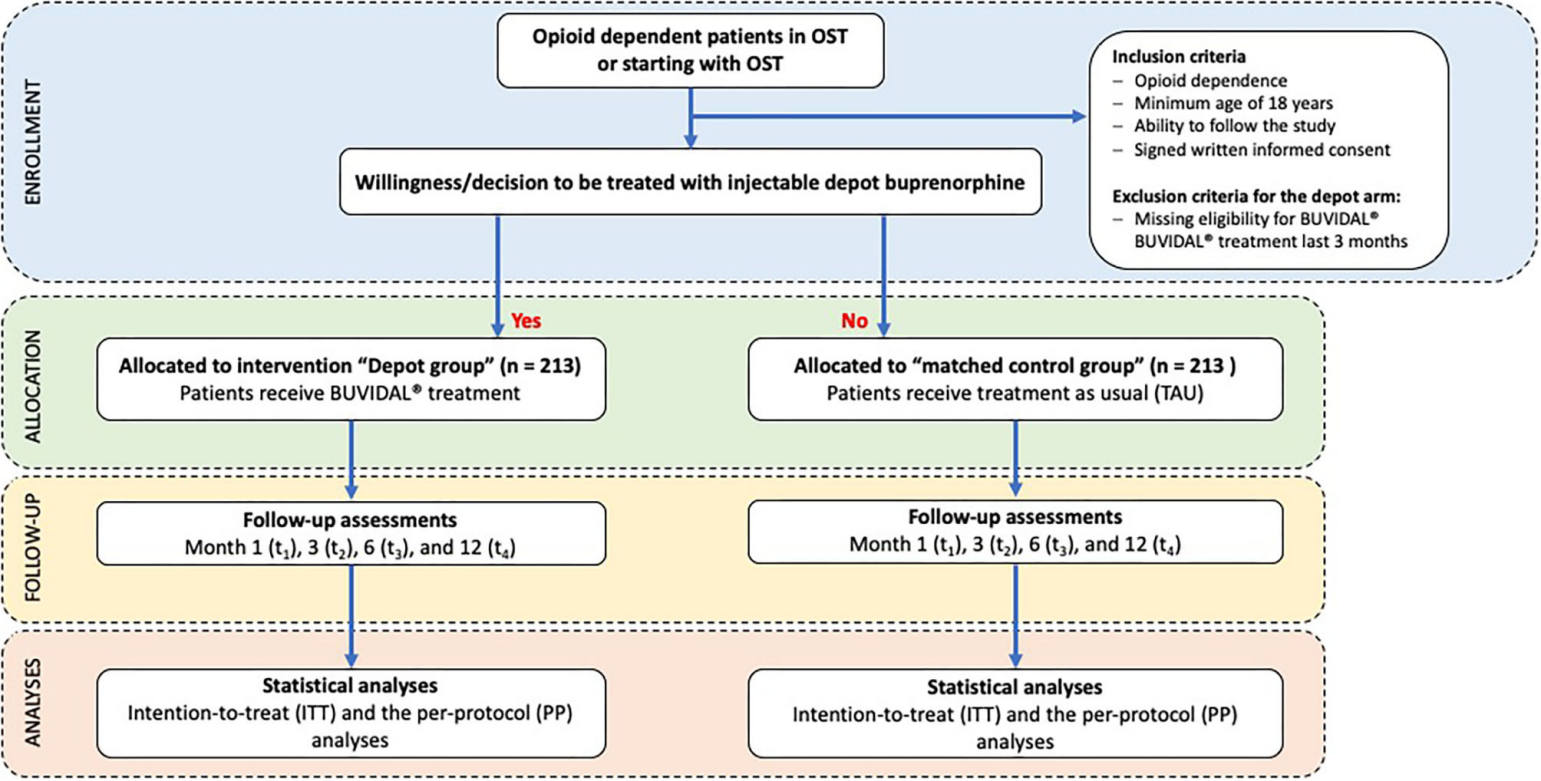
CONSORT diagram on enrollment, allocation, follow-up and analyses.

The study objectives will be evaluated in a non-randomized prospective non-interventional observational study with control group design (treatment-as-usual, TAU). All OST patients with TAU (i.e., treated with any registered OST medication, but not with injectable depot buprenorphine (e.g., racemic methadone, levomethadone, sublingual buprenorphine or slow release oral morphine) can take part in the control group. In order to avoid a selection bias and to ensure an overall comparability of the patient groups (injectable depot buprenorphine vs. TAU), one suitable patient from the OST unit will be pairwise matched to each recruited depot buprenorphine patient. Matching variables are gender (male /female), duration of OST (years), take-home prescription (yes /no) and patients' functioning measured by the global assessment of functioning scale (according to percentiles measured by the Global Assessment of Functioning Scale, GAF). Prior to matching, all patients from each participating OST unit will be screened for these four criteria based on an anonymized list of patients. After inclusion of a depot buprenorphine patient, a respective OST patient who is not willing to switch to depot treatment will be asked to participate as control group patient. An overview of the study design is provided in Figure 1.

### Primary Outcome Measure

The **primary endpoint** of the study is the change in quality of life measured by the difference of OSTQOL total score between baseline (t_0_) and month 12 (t_4_) in the depot buprenorphine group.

### Secondary Outcome Measures

Secondary endpoints include the difference in the OSTQOL score between depot and control group at month 12 (t_4_), comparisons between the depot and control group in patient satisfaction (Client Satisfaction Questionnaire–CSQ-8) at month 12 (t_4_), substance use (i.e., on the basis of self-reporting) at month 6 (t_3_) and month 12 (t_4_), the retention rate in treatment at month 12 (t_4_), psychological distress (Brief Symptom Inventory 18) at t_4_, social participation at month 6 (t_3_) and month 12 (t_4_) as well as the cost-effectiveness (on the basis of quality-adjusted life years (QALYs); EuroQOL five dimensions questionnaire (EQ-5D) at month 12 (t_4_). Three secondary endpoints will be measured solely in the depot group, the acceptance of the depot medication and its application measured by the Treatment Satisfaction Questionnaire for Medication (TSQM-14) at month 1, 3, 6 and 12 (t_1_-t_4_), the dosage adequacy measured by the Opiate Dosage Adequacy Scale (ODAS) at month 1, 3, 6 and 12 (t_1_-t_4_) and changes in quality of life domains (OSTQOL subscales) in depot group over time between baseline (t_0_) and month 12 (t_4_). Possible adverse reactions will be assessed by a physician and recorded as part of the documentation of adverse events (AEs) and adverse drug reactions (ADRs). An overview of all primary and secondary outcome measures is provided in Table 1.

**Table 1:** Overview of primary and secondary outcome measures.

**Primary outcome measure**	**Difference of OSTQOL total score in depot group between baseline (t_0_) and month 12 (t_4_)**
**Secondary outcome measures**	1. Quality of life (OSTQOL score) between depot and control group at t_4_ 2. Acceptance (TSQM-14) of injectable depot buprenorphine between t_1_ and t_4_ 3. Patient satisfaction with OST care (CSQ-8) between depot and control group at t_1_- t_4_ 4. Dosage adequacy (ODAS) in depot group at t_1_-t_4_ 5. Substance use (i.e., self-reporting) between depot and control group at t_3_ and t_4_ 6. Psychological distress (BSI-18) between depot and control group at t_4_ 7. Social participation between depot and control group at t_4_ 8. Retention rate (percentage of patients) in depot group at t_3_ and t_4_ 9. Cost-effectiveness [on the basis of quality-adjusted life years (QALYs); EQ-5D] between depot and control group at t_4_ 10. Changes in quality of life domains (OSTQOL subscales) in depot group between baseline (t_0_) and month 12 (t_4_)

*t_0_, baseline; t_1_, month 1; t_2_, month 3, t_3_, month 6 and t_4_, month 12; OSTQOL, Opioid Substitution Treatment Quality of Life scale; TSQM-14, Treatment Satisfaction Questionnaire for Medication; CSQ-8, Client Satisfaction Questionnaire; ODAS, Opiate Dosage Adequacy Scale; EuropASI, European Addiction Severity Index; BSI-18, Brief Symptom Inventory 18; CRF = Case-Report Form; EQ-5D, EuroQOL five dimensions questionnaire*.

### Variables

The following variables will be collected at baseline (t_0_) and follow-ups [month 1 (t_1_), 3 (t_2_), 6 (t_3_), 12 (t_4_)]:

- **Sociodemographic data** including gender, age, current housing, work, family status/partnership.
- **Health data** will be collected using the Opiate Treatment Index-Health Symptom Scale (OTI-HSS), a symptom checklist to assess the patient's current health status with regard to medical problems that opioid users often develop (e.g., injection-related problems, cardio/respiratory problems, musculoskeletal problems, neurological problems, gastro-intestinal problems (21, 22).
- **OST data** (medication, doses) routinely collected by the OST practice/clinic.
- **Quality of life** will be measured by the OSTQOL, a disease-specific instrument for measuring quality of life among patients in OST (20). First analyses in developmental processes of the OSTQOL showed acceptable internal consistencies of all subscales (personal development, psychological stress, social contacts, material well-being, OST and discrimination), with Cronbach's alpha between 0.75 and 0.88, as well as a good convergent and discriminant validity of the instrument (20).
- The **patient acceptance** of injectable depot buprenorphine will be measured along the four key dimensions of the Treatment Satisfaction Questionnaire for Medication (TSQM-14) (effectiveness; side effects; convenience and global satisfaction).
- The **Client Satisfaction Questionnaire (CSQ-8)** will be used to measure the patients' satisfaction with the overall OST care. The short 8-item instrument has been frequently used in substance use research including OST (23, 24) and has shown a high internal consistency and concurrent validity (25).
- Parts (heroin craving, acceptance of the OST medication) of the **Opiate Dosage Adequacy Scale (ODAS)** will be used to measure the adequacy of opioid doses among the patients in the depot buprenorphine group. The ODAS consists of six domains (heroin consumption, narcotic blockade, mental and somatic opiate withdrawal syndrome, craving for heroin, and OST overdosing) including items which can be answered by the patient. Scores range between 1 (worst) and 5 (best). Cut-off scores of 3 or less in any ODAS domain will be regarded as not adequately dosed. The ODAS was found to have a good validity and reliability to measure and assess buprenorphine dose adequacy in the context of OST (26).
- Data on **substance use** will be collected on the basis of patient self-report using the drug and alcohol use domain of the European Addiction Severity Index (EuropASI) (27). The data on substance use will be complemented by urine samples collected within routine OST care. The urine samples will be tested for heroin, non-prescribed substitutes, cocaine/crack, amphetamine, cannabis and benzodiazepine use.
- **The psychological distress** among the patients will be measured by the Brief-Symptom-Inventory 18 (BSI-18), (28). The questionnaire comprises 18 items on physical and psychological symptoms, rated on a 5-point Likert scale (0 = not at all; 4 = very much) with regard to the last 7 days, and yields 3 sub-scores for depression, anxiety, and somatization, as well as an overall Global Severity Index (GSI).
- **Social participation** will be measured by a self-developed short questionnaire including questions on the number and kind of social activities (e.g., recreational or physical activities, activities with friends, family) within the last week.
- Data to calculate the **retention in treatment** will be collected by the date of ending OST or changing OST medication. The dates will be collected retrospectively by the treatment staff at any follow-up timepoint of the study. Retention in treatment will be calculated by dividing the number of patients in each study arm at each follow-up visit by the total number of patients in each study arm at baseline.
- Quality-adjusted life years (QALYs) will be used for the **cost-effectiveness analysis**. Respective data will be collected with the EuroQOL five dimensions questionnaire (EQ-5D) (29).

### Study Duration

The study duration for each patient will be 12 months, starting with the first dose of depot buprenorphine and ending with the last follow-up assessment at month 12 (t_4_). For matched control patients, the study starts with their baseline examination (after informed consent) and finishes with their participation at the 12-months-assement (t_4_).

Patient recruitment started in April 2020 with first-patient-first-visit. Patients will be recruited over a period of 18 months ending with last-patient-first-visit in September 2021. The individual patient data collection will take further 12 months (last-patient-last-visit in September/October 2022). The data collection, entry and processing of the last incoming CRFs and patient information, the scientific evaluations as well as the production of the final study report will require further six months. Thus, the study will be completed in March 2023 with a total study duration of 39 months.

### Selection and Treatment of Participants

Inclusion and exclusion criteria are listed below. Patients who are not eligible for the treatment with injectable subcutaneous depot buprenorphine according to Summary of Product Characteristics (SmPC) will be excluded from the depot buprenorphine arm, but can be part of the control group, if pairwise matching criteria apply. If a patient drops out from treatment with injectable depot buprenorphine (and returns to previous medication or give up OST at all) he or she should be followed up for the whole study period (unless he/she withdraw the study consent).

#### Inclusion Criteria

##### For both arms

- Opioid dependence (F11.2) according to ICD-10
- Minimum age of 18 years
- Ability to follow the study procedures
- Signed written informed consent. The approved informed consent form will adhere to the ethical principles that have their origin in the Declaration of Helsinki.

##### For the depot buprenorphine arm

- Willingness to be treated with injectable subcutaneous depot buprenorphine.

#### Exclusion Criteria

##### For both arms

- Inability of the patient to participate in the study (e.g., due to severe mental impairment)
- Missing patient-signed written informed consent (this means that patients who cannot give their consent will be excluded, even if their legally acceptable representatives provide the willingness of the patient to participate).

##### For the depot buprenorphine arm

- Missing eligibility for OST with injectable subcutaneous depot buprenorphine according to (SmPC)
- OST with injectable subcutaneous depot buprenorphine within the last 3 months.

### Patient Recruitment

The study will be carried out in OST units (specialized primary care practices or addiction outpatient clinics) with OST physicians experienced in the substitution treatment of opioid-dependent patients. All patients who start OST with injectable depot buprenorphine or who have the intention to switch from a previous OST medication to injectable depot buprenorphine will be informed by the OST physician about the possibility to participate in the study. The decision to be treated with injectable depot buprenorphine has to be made in advance and independently of study participation. All patients who fulfill the inclusion criteria will receive the detailed study information. The matching and recruiting process will be documented by lists, including the assignment of the patient and the code number. The recruitment lists will be kept in the OST unit and will be treated confidential. Only staff from the OST unit will have access to the recruitment lists. The inclusion of a patient will be documented on a specific form and will be faxed or sent as a scan to the study center (ZIS).

### Treatment

The depot arm of the study will include opioid-dependent patients who are willing to switch or to initiate OST with one of two injectable depot buprenorphine formulations (once weekly or once monthly). Thus, this includes newly admitted patients who are starting with OST, as well as patients who are already in treatment (e.g., with racemic methadone, levomethadone, sublingual buprenorphine, slow release oral morphine). The participation in the study is voluntary and non-participation will not incur disadvantages neither for the physician (or the OST unit) nor for the patient. In this respect, there will be no increased risk resulting from study participation. Patients who were facing problems with their previous OST medication might benefit from study participation, and probably show an increased acceptance and willingness to use subcutaneous, injectable depot buprenorphine. Further, OST providers might benefit from expanded, more individualized therapeutic options for OST patients. Based on the clinical experiences gained so far, injectable depot buprenorphine is highly accepted by patients, and its side effect profile is comparable to sublingual buprenorphine with few, mild and reversible injection site reactions (17).

### Assessment and Timelines

All data will be collected in case-report-forms (CRFs) including documentation sheets for clinical routine data and questionnaires for OST providers and patients. After baseline (t_0_), follow-up assessments will be made on month 1 (t_1_), month 3 (t_2_), month 6 (t_3_), and month 12 (t_4_). For the case that OST ends prematurely (before the end of the 12-month study period), study participation continues, and the patient will be asked to take part in the remaining follow-up assessments. All patients will receive reimbursements of € 10,-for each assessment.

### End of Study

After 12 months, with the date of the corresponding documentation and patient survey, the study will end for each patient. As the study participation is voluntary, the patient can prematurely terminate the study at any time. After the study, OST will be continued as needed. Neither study participation nor study termination will have implications on any treatment condition or on the future course of OST.

### Data Analysis

#### Study Sample

Patients will be described (if necessary separated by gender) according to their baseline characteristics and OST data. For categorical data relative frequencies, for continuous data means and standard deviations will be applied.

#### Primary Outcome Analysis

The main analysis will be carried out according to the intention-to-treat principle (ITT), considering all patients who have started the substitution treatment with injectable depot buprenorphine. The primary outcome measure is the difference of OSTQOL (20) scores between baseline (t_0_) and month 12 (t_4_) in the depot group. The average OSTQOL mean score will be compared between these time points and analyzed for statistical significance using dependent *t*-tests. It is tested against the statistical null hypothesis (H0) of a decrease or no change in the OSTQOL mean score. The alternative hypothesis (H1) consists in a significant improvement of the OSTQOL mean score, which corresponds to an improvement in quality of life of the patients.

H0: M (Month 12) ≤ M (baseline) (OSTQOL mean score at 12 months ≤ baseline OSTQOL mean score)
H1: M (Month 12) > M (baseline) (OSTQOL mean score at 12 months > baseline OSTQOL mean score).

The hypothesis will be tested two-sided at a significance level of *p* < 5%. The statistical analyses will be made according to the intention-to-treat (ITT) as well as to the per-protocol (PP) principle. The PP sample is defined by all patients who have undergone OST with injectable subcutaneous depot buprenorphine for at least 12 months and for whom sufficient baseline and 12-month data are available. The ITT sample comprises all patients who provided a written consent, who have undergone the baseline assessment, and–for patients in the depot study arm–who have received the first dose of injectable depot buprenorphine. For the ITT analysis, data from all study patients will be used independently of their status in or out of treatment with injectable depot buprenorphine. Missing values at the 12-month time point will be replaced by missing imputation procedures. These may either include last observation carried forward (LOCF) approaches or longitudinal mixed model analyses using all available follow-up assessments. The choice of imputation procedures for the ITT analysis will depend on the proportion of missing values at the 12-month time point as well as the quantity of available data at 3-month and 6-month follow-up, and will be determined by sensitivity analyses.

#### Secondary Outcome Analyses

Secondary data analyses will be carried out in accordance with the scale levels of the variables or scores and indices (drug consumption, somatic or mental health symptoms, quality of life, withdrawal symptoms, craving, treatment satisfaction) and along the expected relationships between individual variables, e.g., in the context of bivariate or multivariate analyses. Comparisons between depot patients and matched control group will be done by a mixed model approach with baseline OSTQOL score and the (pairwise) cluster term as covariates and will include all follow-up measurement points, such as month 3 and month 6 (see Table 2). Retention rates will be analyzed using descriptive statistics. For group comparisons, Chi^2^-tests (percentages) as well as Kaplan-Meier survival methods and Log-Rank Tests will be used. The significance level will be defined at *p* < 5%.

**Table 2:** Overview of outcome domains, instruments and measurement.

**Outcome domain**	**Instrument**	**t_**0**_**	**t_**1**_**	**t_**2**_**	**t_**3**_**	**t_**4**_**
Quality of life	OSTQOL	x		x	x	x
Acceptance	TSQM-14	x	x	x	x	x
Patient satisfaction with	CSQ-8	x	x	x	x	x
OST care						
Dosage adequacy	ODAS	x	x	x	x	x
Substance use	EuropASI, Urine samples	x	x	x	x	x
Psychological distress	BSI-18	x			x	x
Social participation	Self-constructed questionnaire	x			x	x
Retention	CRF		x	x	x	x
Cost-effectiveness	EQ-5D	x			x	x
Health	OTI-HSS	x			x	x
Adverse events	AE, SAE, ADR	If necessary				

*t_0_, baseline; t_1_, month 1; t_2_, month 3, t_3_, month 6 and t_4_, month 12; OSTQOL, Opioid Substitution Treatment Quality of Life scale; TSQM-14, Treatment Satisfaction Questionnaire for Medication; CSQ-8, Client Satisfaction Questionnaire; ODAS, Opiate Dosage Adequacy Scale; EuropASI, European Addiction Severity Index; BSI-18, Brief Symptom Inventory 18; CRF = Case-Report Form; EQ-5D, EuroQOL five dimensions questionnaire; OTI-HSS, Opiate Treatment Index-Health Symptom Scale; AE, adverse event; SAE, serious adverse event; ADR, adverse drug reaction*.

#### Safety Analysis

Adverse events (AEs) will be documented by medical OST staff. An AE is defined as any new untoward medical occurrence or worsening of a pre-existing medical condition in a clinical investigation subject administered study drug and that does not necessarily have a causal relationship with this treatment. An AE can therefore be any unfavorable and unintended sign (such as an abnormal laboratory finding), symptom, or disease temporally associated with the use of study drug, whether or not considered related to the study drug. The causal relationship to study drug is determined by a physician and should be used to assess all AEs.

A serious AE (e.g., life-threatening overdose) or possible other adverse drug reactions will be documented in the “Report on adverse drug reactions (ADRs)”. Analyses of safety outcomes will equally be conducted for both samples (PP and ITT).

### Sample Size Calculation

A study by Apelt et al. (30) showed a significant improvement in health-related quality of life (measured by SF-36) with an average effect size of *d* = 0.40 after 12 months among patients who switched from (levo-) methadone or buprenorphine to buprenorphine/naloxone. A study by Karow et al. (31) found effect sizes in the improvement of quality of life measured by MSQOL in patients treated with diamorphine between *d* = 0.73 (after 6 months) and *d* = 0.80 (after 12 months).

An α-error of 5% and a statistical power of 80% (β-error = 20%) will be used. In our study we anticipate a smaller effect size of d = 0.25 which will result in a minimum number of *N* = 128 patients. Further we estimate a drop-out rate of 40% until month 12 (t_4_) resulting in a sample size of *N* = 213 patients treated with injectable depot buprenorphine. Considering the same number of patients in the matched control group, the total sample size is *N* = 426. Under the conditions of an α-error of 5% and a statistical power of 80% this sample size allows for detection of statistically significant differences in group comparisons with an effect size of *d* = 0.35 or higher.

### Data Processing, Missing Data

The completion of the data sets is the responsibility of the OST physician and OST unit staff, who carry out the baseline and follow-up documentation and administer the patient questionnaires at the indicated study timepoints. Before study start, all OST physicians and OST unit staff will be trained in recruitment, study conditions, and data documentation. Within the study, all sheets documented by OST staff will be monitored for plausibility and completeness.

All data will be recorded continuously and checked for completeness, plausibility and correctness. For the evaluation of instruments and scales, the respective rules to handle missing data will be considered. Relevant missing clinical data will be requested by queries. Non-retrievable data become “missing data,” which can possibly be corrected in the context of the evaluation on the basis of substantive plausibility checks, so-called “self-evident corrections.”

Data entry and adjustments are carried out by study center (ZIS) staff who are not involved in the study coordination, monitoring or documentation. The statistical analyses will be done by using the statistics program SPSS 25 (32).

### Drop-Outs

Patients who cannot be analyzed (i.e., missing baseline and follow-up data, not starting treatment with injectable depot buprenorphine) will be excluded from the data analyses. If either the medication with injectable depot buprenorphine or OST treatment or study participation (or both) is discontinued, the reason for the termination or circumstance will be documented. Patients who change from the depot group to the control group or vice versa will be followed-up as long they withdraw their study participation or terminate OST (drop-out). In both cases the reasons will be documented. A drop-out analysis will be performed to determine potential drop-out criteria.

## Discussion

The primary objective of this non-randomized prospective observational study is to evaluate the effects of subcutaneous injectable depot buprenorphine on the quality of life of opioid dependent patients in routine OST care in Germany. Together with the secondary objectives, the study aims to provide an overall assessment toward addiction recovery among OST patients treated with subcutaneous injectable depot buprenorphine. The main strength of the study and added value to previous research is this holistic approach in the study objectives and respective variables. By combining clinical OST routine care data with patient-reported outcomes, and comparing depot buprenorphine against other OST medications, this study will help to estimate the impact of depot buprenorphine on quality of life and recovery under real-world conditions.

At the same time, this naturalistic design bears some limitations, most importantly the lack of randomization. OST patients with the decision to switch to subcutaneous injectable depot buprenorphine might differ from other OST patients in several aspects, such as disease severity and psychosocial functioning. To minimize potential selection biases, a matched pair control group design was selected to ensure that OST patients in the depot buprenorphine group will not significantly differ from those in the control group with respect to gender, duration of OST, take-home prescription and psychosocial functioning (GAF-percentiles). A second advantage of the matched-pair design is that OST units in Germany are organized in different primary care settings and vary in the provision of health and psycho-social care as well as in the number of patients per unit. As the number of OST patients in the depot buprenorphine group per unit might be low, a pairwise matching of control patients from the same OST unit was selected instead of a cluster-randomized controlled trial design with a fixed number of patients per cluster.

Another limitation consists in the heterogeneity of the TAU group, especially in terms of OST medication. A comparison between depot buprenorphine and sublingual buprenorphine only would help to identify effects of the application form. However, as (levo-)methadone is still the most frequently used OST agent in Germany (BfArM 2020) (2), we preferred not to exclude this large patient group and adhere to our naturalistic and observational approach.

One further limitation is that the study duration for each patient (12 months) only allows to measure recovery processes over a short period of time. As addiction recovery is a lifelong process, the study therefore does not provide empirical data about the processes of recovery over a longer period of time.

Altogether, this study will provide new insights in the addiction recovery processes of opioid-dependent patients in OST treated with depot buprenorphine over time, including comparisons with patients treated with other OST medications.

## Ethics and Dissemination

### Ethical Considerations

In accordance with § 67 of the AMG (Arzneimittelgesetz, Medicinal Products Act) the study has been announced at the Federal Institute for Drugs and Medical Devices (Bundesinstitut für Arzneimittel und Medizinprodukte, BfArM), the National Association of Statutory Health Insurance Physicians (Kassenärztliche Bundesvereinigung, KBV), the National Association of Statutory Health Insurance Funds and the Private Health Insurance Association.

The study and its documents (including study protocol, case-report-form, patient information, patient consent) has been approved by the independent ethics committee (IEC) of the Hamburg Chamber of Physicians (Ärztekammer Hamburg, reference number: PV7078; September 19^th^, 2019). Upon receipt of the primary ethical vote, secondary votes will be applied for OST units outside of Hamburg.

The study will be conducted in accordance with the current version of the Declaration of Helsinki (adopted by the 18th General Assembly of the World Medical Association in Helsinki, Finland, in June 1964 and supplemented by the 29th General Assembly in Tokyo, Japan, in October 1975, 35th General Assembly in Venice, Italy, October 1983, 41st General Assembly in Hong Kong in September 1989, 48th General Assembly in Somerset West, Republic of South Africa, October 1996 and the 52nd General Assembly in Edinburgh, Scotland, in October 2000, and the clarifications, made by the 53rd General Assembly of the World Medical Association in Washington, USA in October 2002 and the 55th General Assembly in Tokyo, Japan, in October 2004, as well as the revisions by the 59th WMA General Assembly in Seoul, Korea, in October 2008 and the 64th WMA General Assembly Fortaleza, Brazil, in October 2013).

#### Patient Information and Informed Consent

To be eligible for study participation, all patients have to provide their written consent after having been informed in detail about the study objectives, nature, extent and risks. The date of the written consent has to be noted. Prior to consent, no investigations, surveys or other measures associated with the study will be conducted.

#### Data Protection

Throughout the documentation and evaluation phases, all patients will be solely identified by an encoding number assigned to them for the purpose of this study. Patients will be informed that all findings and data collected during the study are stored electronically and treated with strict confidence.

#### Liability and Insurance

As non-interventional and observational, the study is not subject of §§ 40 and 41 of the German Federal AMG and therefore does not require a patient insurance. Participation in the study will not expose the patients to any particular risk.

#### Quality Assurance

As part of the quality assurance, the OST unit staff will be briefed on the study objectives and the study documentation. On-site monitoring will be conducted. Within the monitoring, trained employees of the Center for Interdisciplinary Addiction Research of Hamburg University (ZIS) will check the collected data for completeness and plausibility. Missing data will be supplemented by the study centers on-site. The study has been registered at the German Register of Clinical Trials (DRKS, ID: DRKS00020797).

#### Handling With AEs and ADRs

The occurrence of adverse events will be documented in pseudonymized form by medical OST staff in the “Documentation sheet on adverse events.” The event is classified according to intensity, frequency, outcome, causal link to the substitution medication and time.

Serious adverse events (e.g., life-threatening overdose or possible other adverse drug reactions) will be documented in the “Report on adverse drug reactions (ADRs),” which has to be sent to the study management (ZIS) within 24 h. Incoming ADR messages will immediately be forwarded to the Ethics Committee. All adverse events and adverse drug reactions will be reported in the final report.

#### Dissemination

The study outcomes will be disseminated by presentations at scientific conferences and in peer-reviewed, open-access publications.

## Ethics Statement

The studies involving human participants were reviewed and approved by Hamburg Chamber of Physicians (Ärztekammer Hamburg) (reference number: PV7078). The patients/participants provided their written informed consent to participate in this study.

## Author Contributions

BS, UV, and JR designed the study. BS, KL, and CS wrote the manuscript. IS, UV, and JR commented on the manuscript. All authors reviewed and approved the final version of the manuscript.

## Conflict of Interest

BS had received an unrestricted educational grant from Camurus. UV received speaker's honoraria and traveling expenses from Mundipharma and received traveling expenses from Camurus. JR had served as consultant for Camurus, Indivior, Mundipharma, Sanofi-Aventis, was member of the speakers bureau for Camurus, Hexal, Indivior, and received unrestricted educational grants from Mundipharma. The remaining authors declare that the research was conducted in the absence of any commercial or financial relationships that could be construed as a potential conflict of interest.
